# A Treatise on Protracted Indigestion, and Its Consequences, Addressed by the Author, on His Retirement from the Medical Profession, Both to the Members of That Profession and to the Well-Educated Public, Particularly Parents

**Published:** 1843-04

**Authors:** 


### Art. XIX.

*A Treatise on Protracted Indigestion, and its Consequences. Addressed by the Author, on his retirement from the medical profession, both to the members of that profession and to the well-educated public, particularly parents.* By A. P. W. Philip, m.d. f.r.s.—*London,* 1842. 8vo, pp. 367.

We have seldom risen from the perusal of any work, with feelings of greater surprise and disappointment than from that of the volume now before us. Regarded in either a literary or a professional point of view, this treatise is by no means worthy of Dr. Philip. The author's reasons for addressing the work to the public are both singular and amusing. After informing us that he could not "leave the profession with an easy mind," without communicating to it the important matters contained in this work, he adds, that, having found his professional brethren so dull as not to attend to his lucubrations, he now lays the facts before the public in order that they may be "led to insist on their physicians affording them the attention which their importance demands." (Pref. p. xviii.) Did it not occur to the Doctor, that besides "the difficulty of changing principles" long acted on, there might be one other reason why the profession generally may not have seen fit to embrace his peculiar views; namely, because those views did not seem deserving of adoption? Besides, why address the "public," when there is constantly rising up a young race of practitioners, whose principles are not yet fixed; who are as yet unwedded to system; and from whom, accordingly, Dr. Philip's lucubrations would have met with dispassionate treatment? We may moreover state our most firm persuasion that this appeal from the profession to the public will be wholly vain. A work more totally unadapted to the comprehension and tastes of non-professional readers, we have never seen. We ourselves have been obliged to abandon in despair the attempt to arrive at the meaning of large portions of it. The style throughout is in the highest degree clumsy, obscure and complicated; and the work seems to have been eked out by passage being added to passage, without any reference to methodical sequence. The book is moreover full of the most tedious repetitions of the same subjects and ideas in precisely the same language.

From the first to the one hundred and twenty-ninth page, the author occupies himself with attempts at illustrating the distinctions between the nervous and sensorial powers; the various species of nervous influence, &c. And we feel it to be our duty to warn every one desirous of obtaining clear and accurate information on these subjects, not to expect it in the work before us. This part of the work consists of a mysteriously and laboriously worded statement of facts and doctrines in neuro-physiology perfectly familiar to tyros in that branch of medical science; mixed with several dogmatic reassertions of antiquated errors. We must call attention to one of these; for it would be unjustifiable in us to let it pass.

Thus at page 12, we find Dr. Philip, at this hour of the day, affirming that the "nervous power or influence," which has "been regarded as peculiar to the living animal," is possessed by "inanimate nature" "in common with" it, i.e. the animal. And in the same page, we are informed that after the "nervous influence has been wholly removed, and its functions in consequence have wholly failed, they are as perfectly performed (!) by voltaic electricity, as if the nervous influence itself had been restored; and as we have no means of distinguishing any power but by its effects, the identity of the effects necessarily implies that of the power, as far as relates to its general nature." Ergo (in other words,) the nervous power and voltaic electricity are identical! And this conclusion, the author tells us (page 12,) has been established by "the most conclusive of all tests, (query, experiments?) which have been publicly repeated both in London and Paris," &c.

Now there is here either the grossest ignorance, or a serious want of candour. Surely Dr. Philip does not require to be informed that the inference drawn so hastily and unphilosophically many years ago, by some experimentalists, that because galvanism or electricity applied to the not-yet dead bodies of animals causes contractions of the muscles, and even resuscitates for a very brief period, and in a very imperfect degree, the flagging or torpid functions of the lungs and stomach, is long since shown to be erroneous; and that the real explanations of the phenomena, is, that in such cases, galvanism or electricity acts simply as a stimulant, analogous to, though perhaps more powerful than friction, or the sudden application of heat or cold, or of certain chemical substances. Although the nerves going to a part or organ are cut across, yet it is well known that the nerves in the separated part, retain for a short time the capacity of being stimulated by mechanical or chemical agents; but it is absurd to conclude that these agents are the final causes of the short-lived actions which they excite.

Moreover, is Dr. Philip not aware that his experiments, bearing on the above subjects, have been repeated, in the way directed by himself, by Müller and Dr. Dieckhof, and with results different from his? (See Baly's Translation, vol. i, p. 550.) Is he ignorant that Breschet and Edwards, after careful experiments, have come to the conclusion, that the restoration of the digestive process, on the application of electricity, is not owing to the electricity itself, but to the irritation of the vagus nerve; and that mechanical irritation produces precisely the same effect? (Archives Génèrales de Médecine, 1828.) If Dr. Philip is aware of these facts, how can he venture to express himself, as if the *identity* of the nervous power and electricity were still the received doctrine of physiologists? If he is *not* aware of these facts, what an amazing and culpable degree of ignorance does this circumstance imply? Yet this alleged identity of the two powers above named is asserted not once, but we should think, a dozen times, in the course of the work!

The grand discovery, to announce which the work seems to have been published, is, that a certain hepatic (Dr. Philip writes it hypatic,) affection, named by the author, the distended liver, is a frequent disorder, and, through the medium of what Dr. Philip calls "a fret of nerves," leads to a formidable and often fatal debility of the brain and spinal cord; the second and third stages of the distended liver corresponding with the first and second of debility of the brain and spinal marrow. We do not find, on a sufficient inspection, of this part of the volume, anything materially new, either as regards diagnosis or treatment. In these respects, the present work seems, to a great extent, but a *rifacimento* of the information contained in the author's Treatise on Indigestion. Here, as there, his pathological views are limited to the consideration of one only of the multifarious forms of affection, to which the organ he is treating of is subject. As in his Treatise on Indigestion, an inflammatory affection of the pyloric end of the stomach is, were we to credit Dr. Philip, the sole source of all dyspepsia; (disorders of the function of innervation, &c. being wholly overlooked, as well as those forms of derangement arising from sympathy with other organs, as the brain, &c.;) so, in the treatise before us, the manifold disorders, incident to the liver, from the fact of its being an excretory organ, from its often acting supplementary of the lungs and skin, &c., are neglected to be even adverted to. In regard to treatment, Dr. Philip here, as in his former work already alluded to, directs small doses of mercury, (1-20th of a grain of calomel,) and advises a seton, which, if we remember aright, he does not make mention of in his former treatise. This seton is to be placed over the region of the liver. As regards other internal means, he recommends hyoscyamus and the extract of poppy as the best sedative in cases of distended liver, and creosote (?) as the best tonic.

Nearly the last half of the work is occupied with another reprint of the papers from the Philosophical Transactions, which we have had so often before! We cannot conclude this notice, without animadverting to the fact of the total non-reference by Dr. Philip to contemporary labourers in the same department of pathology, namely, dyspeptic disease. No allusion whatever is made in the volume before us, to the works of Drs. Paris, Johnstone, Combe, Dick, Langston Parker, &c.: yet in one or other of these, is contained all the useful information to be found in Dr. Philip's work, with a great deal not to be found there.

